# Thermal Effects of Rapid High‐Intensity Light Curing on Bulk‐Fill Resin‐Based Composites: A Systematic Review and Meta‐Analysis

**DOI:** 10.1155/tswj/5519049

**Published:** 2025-12-28

**Authors:** Samille Biasi Miranda, Marina Rodrigues Santi, Giovana Lordsleem de Mendonça, Luiz Antonio Soares Falson, Matheus José Gusmão Simões Barza, Veronica Maria de Sá Rodrigues, Ana Karina Maciel de Andrade, Rodrigo Barros Esteves Lins, Marcos Antonio Japiassú Resende Montes

**Affiliations:** ^1^ Departament of Dental Materials, School of Dentistry, University of Pernambuco, Recife, Pernambuco, Brazil, ufpe.br; ^2^ Departament of Restorative Dentistry, Piracicaba Dental School, University of Campinas, Piracicaba, São Paulo, Brazil, unicamp.br; ^3^ Department of Semiology and Clinical, Faculty of Dentistry, Federal University of Pelotas, Pelotas, Rio Grande do Sul, Brazil, ufpel.edu.br; ^4^ Departament of Restorative Dentistry, School of Dentistry, Federal University of Paraíba, João Pessoa, Paraíba, Brazil, ufpb.br; ^5^ Departament of Restorative Dentistry, School of Dentistry, Federal University of Alagoas, Maceió, Alagoas, Brazil, ufal.edu.br

**Keywords:** dental curing lights, high irradiance, resin-based composite, thermal effects

## Abstract

**Objective:**

The objective of this study is to evaluate whether high‐intensity, short‐duration light curing of bulk‐fill resin‐based composite (RBC) causes an increase in temperature of the material, compared to the standard light‐curing protocol.

**Methods:**

This review was performed in accordance with the Preferred Reporting Items for Systematic Reviews and Meta‐Analyses statement and registered in the Open Science Framework database (10.17605/OSF.IO/UNW7C). Electronic searches were carried out in the PubMed/MEDLINE, Embase, Web of Science, Scopus, and Virtual Health Library databases for articles published up to April 2025. In vitro studies comparing the increase in temperature during high‐intensity light curing and the standard protocol for bulk‐fill RBCs were considered eligible. Seven different parameters assessed the risk of bias, and the studies were subjected to two 2 meta‐analyses (light curing of 3 and 10 s and 3 and 20 s), according to the increment thicknesses (1–4 mm in depth) of the bulk‐fill RBCs. The quality of the evidence was assessed using the GRADE tool.

**Results:**

The search identified 607 studies. After applying the eligibility criteria, six studies were included in the review, with one study classified as having a moderate risk of bias and five studies classified as high risk. Four studies were included in two different meta‐analyses, which presented moderate heterogeneity (*I* ^2^ = 56*%*, 88%, and 66%, respectively). The first meta‐analysis (comparing 3 and 10 s light‐curing protocols) showed statistical significance (*p* = 0.008), while the second meta‐analysis (comparing 3 and 20 s) did not demonstrate statistical significance (*p* = 0.20). The certainty of the evidence was rated as very low.

**Conclusion:**

The use of high‐intensity, short‐duration light‐curing protocols is thermally applicable based on limited in vitro studies with very low certainty of evidence. However, in clinical situations involving deep cavities with reduced residual dentin thickness, the use of high‐intensity curing should be avoided to minimize the risk of thermal damage.

## 1. Introduction

Resin composites are the most widely used materials for direct restorations [1]. The conventional technique involves incremental layering of 2 mm, but this approach is time‐consuming and increases the risk of procedural errors, such as contamination and air entrapment, potentially leading to marginal gaps, voids, and postoperative sensitivity [2, 3]. To overcome these limitations, bulk‐fill resin‐based composites (RBCs) were introduced, allowing increments of up to 4–5 mm, thereby simplifying the restorative procedure while improving mechanical properties, reducing polymerization stress, and enhancing marginal integrity [3–7].

Recent advances have further aimed to decrease clinical time through the use of high‐intensity light‐curing protocols, utilizing light‐curing units (LCUs) capable of delivering irradiance levels above 3000 mW/cm^2^, enabling effective polymerization within 3 s [8]. To support this approach, *β*‐allyl sulfone (AFCT) technology was incorporated into the resin matrix of specifically developed bulk‐fill composites, enhancing their thermal resistance and enabling ultrafast curing without compromising mechanical properties or the degree of conversion [9]. However, despite these benefits, concerns have arisen regarding the potential thermal effects of rapid, high‐intensity polymerization on dental tissues, particularly in deep cavities where the remaining dentin thickness is reduced [10].

Temperature increases during the light‐curing process are clinically significant because excessive heat generation can lead to irreversible pulp damage if critical thresholds are surpassed. Zach and Cohen [11] demonstrated that a temperature rise of 5.5°C or more within the pulp chamber can result in permanent pulpal injury. Moreover, bulk‐fill restorations may lead to increased intrapulpal temperature rise during polymerization, particularly due to the larger volume of material and the use of high‐intensity LCUs, highlighting the need for thorough evaluation of thermal effects in such protocols [12].

Given these considerations, this systematic review and meta‐analysis are aimed at investigating whether high‐intensity, short‐duration light curing results in a greater temperature increase in bulk‐fill RBCs compared to conventional light‐curing protocols. The null hypothesis was that high‐intensity light curing would not produce a higher temperature rise relative to standard light‐curing procedures.

## 2. Materials and Methods

### 2.1. Registration and Protocol

This systematic review was conducted in accordance with the Preferred Reporting Items for Systematic Reviews and Meta‐Analyses (PRISMA) guidelines [13] and was registered in the Open Science Framework (OSF) database under doi:10.17605/OSF.I/O/UNW7C.

### 2.2. Eligibility Criteria

The inclusion of studies in this systematic review was based on the population, intervention, comparison, and outcome (PICO) strategy: The population consisted of bulk‐fill RBCs; the intervention was high‐intensity, short‐duration light curing of bulk‐fill RBCs; the comparison was the standard light‐curing protocol of bulk‐fill RBCs; and the outcome evaluated was the temperature change resulting from the light‐curing protocol. The guiding research question was as follows: “Does high‐intensity, short‐duration light curing cause a greater increase in temperature when compared to the standard protocol in bulk‐fill RBCs?”

Only in vitro experimental studies assessing temperature changes in RBCs under high‐intensity versus standard curing were included. Exclusions comprised animal studies, case reports, abstracts, expert opinions, and studies using bulk‐fill RBCs as a base material or evaluating insertion techniques of other restorative materials.

### 2.3. Sources of Information and Search Strategy

Two independent reviewers (S.B.M. and M.R.S.) searched for articles published up to April 2025 in the electronic databases PubMed/MEDLINE, Embase, Web of Science, Scopus, and Virtual Health Library. No date or language restrictions were applied. A search strategy was initially carried out in the PubMed/MEDLINE database and was then adapted to the other databases, as shown in Table 1. Additionally, a manual search analysis was conducted by screening the reference lists of the included articles to identify other eligible studies.

**Table 1:** Search strategy for each electronic database.

**Databases**	**Search strategy**	**Filter**
PubMed/MEDLINE	(Resin composite bulk fill) OR (Bulk fill) OR (Bulk fill composite) OR (Bulk fill composite resin) OR (Bulk fill resin‐based) OR (Tetric powerfill) OR (Tetric powerflow) AND (Dental curing lights) OR (Light curing) OR (Polymerization) OR (High‐irradiance) OR (High‐power curing light) OR (Ultra‐fast light‐curing) OR (Rapid high‐intensity light‐curing) OR (Shortened light‐curing) AND (Temperature) OR (Temperature rise) OR (Temperature Change) OR (high temperature) OR (Thermal rise) OR (Polymerization temperature) OR (Pulpal temperature) OR (Heating) or (Heat generation)	No filters applied
Embase	(“resin”/exp OR resin) AND (“composite”/exp OR composite) AND (“bulk”/exp OR bulk) AND fill OR ((“bulk”/exp OR bulk) AND fill) OR ((“bulk”/exp OR bulk) AND fill AND (“composite”/exp OR composite)) OR ((“bulk”/exp OR bulk) AND fill AND (“composite”/exp OR composite) AND (“resin”/exp OR resin)) OR ((“bulk”/exp OR bulk) AND fill AND “resin based”) OR ((“tetric”/exp OR tetric) AND powerfill) OR ((“tetric”/exp OR tetric) AND powerflow) AND (“dental”/exp OR dental) AND (“curing”/exp OR curing) AND lights OR ((“light”/exp OR light) AND (“curing”/exp OR curing)) OR “polymerization”/exp OR polymerization OR “high irradiance” OR (“high power” AND (“curing”/exp OR curing) AND (“light”/exp OR light)) OR (“ultra fast” AND “light curing”) OR ((“rapid”/exp OR rapid) AND “high intensity” AND “light curing”) OR (shortened AND “light curing”) AND “temperature”/exp OR temperature OR ((“temperature”/exp OR temperature) AND (“rise”/exp OR rise)) OR ((“temperature”/exp OR temperature) AND (“change”/exp OR change)) OR (high AND (“temperature”/exp OR temperature)) OR (thermal AND (“rise”/exp OR rise)) OR ((“polymerization”/exp OR polymerization) AND (“temperature”/exp OR temperature)) OR (pulpal AND (“temperature”/exp OR temperature)) OR “heating”/exp OR heating OR ((“heat”/exp OR heat) AND (“generation”/exp OR generation))	No filters applied
Web of Science	ALL = ((Resin composite bulk fill) OR (Bulk fill) OR (Bulk fill composite) OR (Bulk fill composite resin) OR (Bulk fill resin‐based) OR (Tetric powerfill) OR (Tetric powerflow)) AND ALL = ((Dental curing lights) OR (Light curing) OR (Polymerization) OR (High‐irradiance) OR (High‐power curing light) OR (Ultra‐fast light‐curing) OR (Rapid high‐intensity light‐curing) OR (Shortened light‐curing)) AND ALL = ((Temperature) OR (Temperature rise) OR (Temperature Change) OR (high temperature) OR (Thermal rise) OR (Polymerization temperature) OR (Pulpal temperature) OR (Heating) or (Heat generation))	No filters applied
Scopus	(TITLE‐ABS‐KEY ((resin AND composite AND bulk AND fill) OR (bulk AND fill) OR (bulk AND fill AND composite) OR (bulk AND fill AND composite AND resin) OR (bulk AND fill AND resin‐based) OR (tetric AND powerfill) OR (tetric AND powerflow)) AND TITLE‐ABS‐KEY ((dental AND curing AND lights) OR (light AND curing) OR (polymerization) OR (high‐irradiance) OR (high‐power AND curing AND light) OR (ultra‐fast AND light‐curing) OR (rapid AND high‐intensity AND light‐curing) OR (shortened AND light‐curing)) AND TITLE‐ABS‐KEY ((temperature) OR (temperature AND rise) OR (temperature AND change) OR (high AND temperature) OR (thermal AND rise) OR (polymerization AND temperature) OR (pulpal AND temperature) OR (heating) OR (heat AND generation)))	No filters applied
Virtual Health Library	((Resin composite bulk fill) OR (Bulk fill) OR (Bulk fill composite) OR (Bulk fill composite resin) OR (Bulk fill resin‐based) OR (Tetric powerfill) OR (Tetric powerflow)) AND ((Dental curing lights) OR (Light curing) OR (Polymerization) OR (High‐irradiance) OR (High‐power curing light) OR (Ultra‐fast light‐curing) OR (Rapid high‐intensity light‐curing) OR (Shortened light‐curing)) AND ((Temperature) OR (Temperature rise) OR (Temperature Change) OR (high temperature) OR (Thermal rise) OR (Polymerization temperature) OR (Pulpal temperature) OR (Heating) or (Heat generation))	No filters applied

### 2.4. Article Selection Process

**Figure 1:**
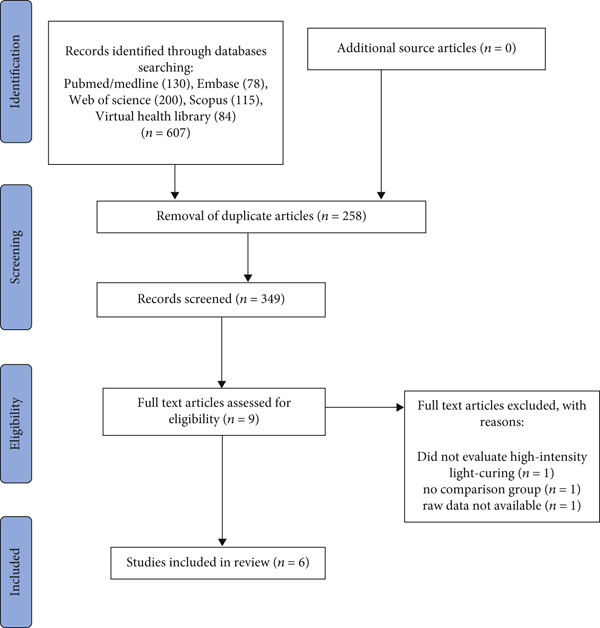
PRISMA flowchart describing study selection.

After retrieving studies from each database, duplicates were removed, and studies were screened using Rayyan Management Software (Qatar Computing Research Institute, Doha, Qatar). Titles and abstracts were initially screened, followed by full‐text review for eligibility. Discrepancies between reviewers were resolved by a third reviewer (G.L.d.M.) through discussion and consensus. The article selection process is summarized in Figure 1. The interexaminer kappa coefficient [20] was used to determine the level of agreement between reviewers, with scores interpreted as 0 (*no agreement*), < 0.8 (*moderate agreement*), or ≥ 0.8 (*almost perfect agreement*).

### 2.5. Data Extraction and Collection Process

Data extraction was performed by one reviewer (S.B.M.) and checked by two others (M.R.S. and G.L.d.M.) to resolve discrepancies. Information collected included study characteristics, sample size, RBC type, curing protocol, temperature assessment method, and main results, organized in a standardized Excel spreadsheet and summarized in tables.

### 2.6. Meta‐Analysis

The extracted data were descriptively synthesized based on relevant variables. Primary outcomes were assessed using the standardized mean difference with 95% confidence intervals. Meta‐analyses were conducted when PICOs were sufficiently homogeneous to yield clinically meaningful results. Two reviewers (S.B.M. and M.R.S.) conducted the analysis, and in case of disagreement, a third reviewer (G.L.d.M.) was consulted.

### 2.7. Risk of Bias Analysis

Risk of bias was evaluated independently by two reviewers (S.B.M. and M.R.S.), based on seven parameters, adapted from a prior systematic review of in vitro studies [21]: (1) randomization of teeth, (2) use of caries‐free teeth, (3) standardization of enamel or dentin surfaces, (4) temperature analysis performed by a single operator, (5) examiner blinding, (6) sample size calculation, and (7) complete data reporting. The checklist was adapted from tools specifically designed for in vitro studies, given the absence of a standardized risk of bias instrument for laboratory studies. When authors reported the parameter, the article presented a “Y” (yes) for that specific parameter. If it was not possible to find the information, the article received an “N” (no). Regarding classification, articles that reported one to three items (Y) presented a high risk of bias, four or five items a medium risk of bias, and six or seven items a low risk of bias. The results are presented in table form.

### 2.8. Quality of Evidence

The quality of evidence was assessed using the Grading of Recommendations, Assessment, Development, and Evaluation (GRADE) approach [22], following the Cochrane Manual for the Development of Systematic Intervention Reviews, Version 5.1.0. GRADE evaluates quality based on study design, inconsistency, indirectness, imprecision, and publication bias. While GRADE is primarily intended for clinical studies, its use in this review was adapted to account for the specific characteristics of in vitro research, such as variability in experimental designs, sample sizes, and temperature assessment methods. The assessment considered the indirectness of the evidence, as the studies were conducted in laboratory settings rather than clinical environments. Results were categorized into four levels: very low, low, moderate, and high. The quality assessment was independently conducted using the GRADE Pro program by two reviewers (S.B.M. and M.R.S.), and any disagreements were resolved by a third reviewer (G.L.d.M.).

## 3. Results

### 3.1. Study Selection

The electronic database search yielded 607 articles: PubMed/MEDLINE (*n* = 130), Embase (*n* = 78), Web of Science (*n* = 200), Scopus (*n* = 115), and the Virtual Health Library (*n* = 84). After removing duplicates, 349 articles remained. Titles and abstracts were screened according to eligibility criteria, resulting in nine articles potentially eligible for full‐text analysis. After reviewing the full texts, three studies were excluded for the following reasons: lack of comparison between high‐intensity and standard light‐curing protocols (*n* = 1), no comparator group of BRCs (*n* = 1), and unavailability of raw data (*n* = 1). Consequently, six in vitro studies were included in the quantitative and qualitative analyses. A schematic flowchart summarizing the article selection process is presented in Figure 1.

The interexaminer agreement for study selection was considered an “almost perfect agreement” with a Cohen′s kappa coefficient of *k* = 0.89.

### 3.2. Characteristics of the Included Studies

Table 2 summarizes the main characteristics of the six included studies [14–19]. All were laboratory‐based experimental studies published between 2021 and 2024. A total of 640 specimens were analyzed, including cylindrical composite samples (*n* = 3), human dentin discs (*n* = 1), and cavities prepared in extracted upper (*n* = 1) and lower molars (*n* = 1). The control group used standard light‐curing protocols, while intervention groups employed high‐intensity light curing. Temperature changes were assessed using prefabricated molds (*n* = 3) or under intradental (*n* = 1) and intrapulpal (*n* = 2) conditions. Temperature was measured using type K, T, and J thermocouples, as well as an infrared thermal camera. Only one study simulated basal pulp temperature.

**Table 2:** Characteristics of the included studies.

**Author, year**	**Design of study**	**n**	**Unit of study**	**Bulk-fill composite resins (experimental)**	**Bulk-fill composite resins (control)**	**Temperature assessment**	**Temperature assessment instrument**	**Oral cavity basal temperature simulation**	**Main results**
Wang et al. (2021) [14]	In vitro	100	Cylindrical composite samples inserted into a 3D‐printed resin mold (5 × 4, 3, 2, and 1 mm)	Tetric PowerFill (Ivoclar Vivadent)	Beautiful Bulk Flow (Shofu), Admira Fusion X‐tra (Voco), and Filtek Bulk Fill Flow (3M/ESPE)	Measurement of the temperature change in real time for every millimeter of the restorative material inside the mold	Five K‐type thermocouples	No	High‐intensity and short‐duration light curing caused a temperature increase.
Yang et al. (2021) [15]	In vitro	60	Dental cavity prepared in the lower first molar, with four measurement locations: (0, 2, and 4 mm from the top of the cavity and 1 mm into the dentin)	Tetric PowerFill and Tetric PowerFlow (Ivoclar Vivadent)	Viscalor (Voco) and Filtek One Bulk Fill (3M/ESPE)	Measuring the intradental temperature change of a molar	Infrared thermal camera with real‐time thermographic measurements	No	High‐intensity, short‐duration light curing was comparable to conventional light curing.
Maucoski et al. (2023) [16]	In vitro	360	Class I (3 × 4 × 5 mm) and V (2 × 2 × 5 mm) cavities prepared in a maxillary molar	Tetric PowerFill and Tetric PowerFlow (Ivoclar Vivadent)	Filtek Bulk Fill Flowable and Filtek One Bulk Fill (3M/ESPE)	Measuring temperature change inside the pulp chamber of a molar	Two T‐type thermocouples	Yes (basal pulp temperature of 32°C)	High‐intensity and short‐duration light curing caused acceptable temperature increases in the pulp chamber.
Miranda et al. (2024) [8, 17]	In vitro	40	Dentin discs (0.5 mm) obtained from human molars embedded in artificial pulp chambers (4.5 × 5 mm)	Tetric PowerFlow (Ivoclar Vivadent)	Filtek Bulk Fill Flowable (3M/ESPE)	Measurement of temperature change using an artificial pulp chamber with human dentin discs	K‐type thermocouple	No	The 3‐s rapid high‐intensity light curing caused greater temperature increases than the standard protocol.
Odum et al. (2023) [18]	In vitro	90	Composite samples inserted into plaster molds with rectangular slots (1.5 × 2 × 7 mm)	Tetric Powerfill (Ivoclar Vivadent)	Filtek One Bulk Fill (3M/ESPE) and Tetric EvoCeram Bulk Fill (Ivoclar Vivadent)	Measurement of the temperature change of 1.5‐mm‐thick samples	J‐type thermocouple	No	High‐intensity and short‐duration light curing caused an increase in temperature.
Thanoon et al. (2024) [19]	In vitro	30	Composite samples inserted into a 3D printed resin mold (4 × 4 mm)	Tetric PowerFill (Ivoclar Vivadent)	Tetric EvoCeram Bulk Fill (Ivoclar Vivadent)	Measuring the temperature change of samples 4 mm deep	Infrared thermal camera with real‐time thermographic measurements	No	Bulk‐fill composite designed for rapid curing showed a higher temperature rise compared to conventional bulk‐fill composite resins.

### 3.3. Temperature Assessment

Table 3 presents the temperature increase results for both the control and intervention groups. Three studies [14, 17, 18] reported a higher temperature increase when bulk‐fill RBCs were light‐cured using greater intensity and shorter exposure time compared to the standard protocol. One study [19] found that bulk‐fill RBCs specifically designed for high‐intensity light curing exhibited significantly greater temperature rises at all tested depths (0, 1, 2, 3, and 4 mm) compared to standard RBCs. In contrast, the only available in vivo study [15] found that 3‐s high‐irradiance light curing produced a comparable temperature rise to standard 10‐s curing. Another study [16] corroborated these findings, reporting that rapid light curing resulted in an acceptable temperature increase within the pulp chamber.

**Table 3:** Assessment of temperature change (*Δ* *T*, °C).

**Author, year**	**Bulk-fill composite resins (experimental)**			**Bulk-fill composite resins (control)**				
	**Exposure condition**	**Mean (SD)**		**Exposure condition**	**Mean (SD)**			
Wang et al. (2021) [14]	Polywave (3 s, 3050 mW/cm^2^)	Resin	PFill	Polywave (3 s, 3050 mW/cm^2^)	Resin	FOB	BBF	AF
		4 mm (top) 3 mm 2 mm 1 mm 0 mm	34.4 (±7.4) 33.1 (±6.4) 32.5 (±6.5) 32.6 (±4.6) 31.6 (±3.7)		4 mm (top) 3 mm 2 mm 1 mm 0 mm	34.6 (±7.7) 35.5 (±7.1) 35.5 (±6.1) 34.3 (±5.3) 30.7 (±3.1)	32.0 (±5.7) 31.5 (±4.9) 30.7 (±3.8) 28.5 (±3.3) 28.7 (±2.3)	31.7 (±5.4) 30.9 (±4.2) 30.9 (±3.9) 29.7 (±2.4) 29.2 (±2.2)
	Polywave (5 s, 2100 mW/cm^2^)	4 mm (top) 3 mm 2 mm 1 mm 0 mm	33.0 (±7.6) 33.8 (±7.4) 32.9 (±5.9) 32.7 (±5.3) 31.6 (±4.1)	Polywave (5 s, 2100 mW/cm^2^)	4 mm (top) 3 mm 2 mm 1 mm 0 mm	34.4 (±6.6) 32.3 (±4.5) 31.3 (±4.0) 33.2 (±3.5) 31.4 (±2.3)	29.5 (±5.8) 29.0 (±4.7) 28.4 (±4.1) 27.6 (±2.7) 27.0 (±2.8)	32.3 (±4.8) 31.9 (±4.7) 31.5 (±3.9) 30.9 (±2.5) 29.6 (±1.7)
	Polywave (10 s, 1200 mW/cm^2^)	4 mm (top) 3 mm 2 mm 1 mm 0 mm	33.4 (±6.2) 33.6 (±5.9) 33.4 (±5.2) 32.9 (±4.7) 31.1 (±2.9)	Polywave (10 s, 1200 mW/cm^2^)	4 mm (top) 3 mm 2 mm 1 mm 0 mm	32.3 (±5.7) 30.2 (±3.5) 31.1 (±3.7) 28.6 (±3.8) 31.4 (±2.7)	29.0 (±5.3) 28.4 (±4.4) 28.6 (±4.0) 27.1 (±2.5) 25.1 (±1.7)	31.5 (±4.2) 30.9 (±4.5) 31.1 (±3.1) 30.0 (±2.5) 29.3 (±2.1)
	Polywave (20 s, 1200 mW/cm^2^)	4 mm (top) 3 mm 2 mm 1 mm 0 mm	35.0 (±7.1) 33.9 (±5.6) 33.7 (±5.0) 32.1 (±4.5) 32.3 (±3.7)	Polywave (20 s, 1200 mW/cm^2^)	4 mm (top) 3 mm 2 mm 1 mm 0 mm	34.6 (±7.7) 35.5 (±7.1) 35.5 (±6.1) 34.3 (±5.3) 33.4 (±4.5)	32.3 (±5.1) 31.6 (±4.7) 30.3 (±3.3) 30.5 (±3.1) 28.2 (±2.0)	34.8 (±5.5) 33.3 (±4.3) 33.4 (±3.7) 29.5 (±1.9) 31.2 (±2.3)
	Monowave (20 s, 1470 mW/cm^2^)	4 mm (top) 3 mm 2 mm 1 mm 0 mm	39.3 (±8.9) 37.4 (±6.2) 36.4 (±5.0) 34.0 (±3.3) 29.0 (±1.7)	Monowave (20 s, 1470 mW/cm^2^)	4 mm (top) 3 mm 2 mm 1 mm 0 mm	38.1 (±7.3) 37.4 (±6.2) 36.5 (±5.0) 34.0 (±3.3) 30.4 (±2.3)	34.6 (±6.1) 36.4 (±6.3) 34.0 (±4.3) 35.2 (±4.3) 27.0 (±1.0)	35.3 (±5.7) 35.3 (±5.6) 34.8 (±4.9) 33.5 (±3.4) 33.0 (±3.4)
Yang et al. (2021) [15]	Bluephase PowerCure (3 s, 3000 mW/cm^2^)	Intradental	PFill	PFlow	Bluephase PowerCure (3 s, 3000 mW/cm^2^)	Intradental	OBF	VC
		0 mm 2 mm 4 mm 1 mm (dentin)	48.5 (0.87) 48.8 (0.40) 41.2 (1.30) 32.9 (0.33)	53.1 (1.94) 54.5 (1.04) 41.2 (3.49) 34.0 (1.51)		0 mm 2 mm 4 mm 1 mm (dentin)	45.8 (1.22) 46.6 (1.07) 38.5 (0.87) 32.3 (0.70)	42.5 (0.86) 43.6 (0.81) 39.1 (1.08) 32.9 (0.56)
	Bluephase PowerCure (10 s, 1200 mW/cm^2^)	0 mm 2 mm 4 mm 1 mm (dentin)	44.0 (1.13) 44.3 (0.72) 38.0 (1.43) 33.7 (0.36)	48.1 (2.23) 50.3 (0.81) 38.8 (0.64) 34.0 (0.53)	Bluephase PowerCure (10 s, 1200 mW/cm^2^)	0 mm 2 mm 4 mm 1 mm (dentin)	44.0 (1.10) 44.3 (0.85) 38.2 (0.61) 33.4 (0.96)	41.0 (0.91) 42.0 (0.72) 38.2 (0.68) 34.2 (0.89)
	Elipar S10 (10 s, 1200 mW/cm^2^)	0 mm 2 mm 4 mm 1 mm (dentin)	47.9 (1.36) 47.9 (0.97) 40.1 (1.25) 35.6 (1.15)	49.3 (1.99) 52.3 (1.88) 39.7(1.26) 35.2 (1.54)	Elipar S10 (10 s, 1200 mW/cm^2^)	0 mm 2 mm 4 mm 1 mm (dentin)	44.5 (2.19) 45.9 (2.33) 37.7 (1.66) 33.6 (1.18)	42.4 (1.49) 43.2 (0.96) 38.5 (1.03) 34.9 (0.64)
Maucoski et al. (2023) [16]	Monet Laser (1 s, 1502 mW/cm^2^) (3 s, 1502 mW/cm^2^	Intrapulpar	PFill	PFlow	Monet Laser (1 s, 1502 mW/cm^2^) (3 s, 1502 mW/cm^2^	Intrapulpar	OBF	FBF
		Class I Class V	1.1 (0.1) 0.7 (0.2)	1.0 (0.0) 0.9 (0.1)		Class I Class V	0.9 (0.2) 0.5 (0.0)	1.2 (0.0) 0.9 (0.1)
		Class I Class V	1.6 (0.1) 0.9 (0.1)	1.9 (0.1) 1.1 (0.2)		Class I Class V	1.5 (0.0) 0.6 (0.0)	2.0 (0.2) 1.0 (0.3)
	PowerCure (3 s, 2818 mW/cm^2^) (20 s, 1057 mW/cm^2^)	Class I Class V	1.4 (0.1) 2.0 (0.1)	1.7 (0.1) 2.2 (0.2)	PowerCure (3 s, 2818 mW/cm^2^) (20 s, 1057 mW/cm^2^)	Class I Class V	1.4 (0.1) 1.9 (0.2)	1.8 (0.1) 2.1 (0.2)
		Class I Class V	2.4 (0.3) 3.3 (0.2)	2.5 (0.1) 4.0 (0.4)		Class I Class V	2.3 (0.2) 3.0 (0.1)	2.7 (0.0) 4.0 (0.3)
	PinkWave (3 s, 1685 mW/cm^2^) (20 s, 1353 mW/cm^2^)	Class I Class V	1.4 (0.0) 1.9 (0.2)	1.5 (0.1) 1.1 (0.1)	PinkWave (3 s, 1685 mW/cm^2^) (20 s, 1353 mW/cm^2^)	Class I Class V	1.2 (0.2) 1.0 (0.0)	1.7 (0.1) 1.2 (0.3)
		Class I Class V	3.7 (0.5) 2.5 (0.3)	3.5 (0.1) 3.3 (0.3)		Class I Class V	3.4 (0.4) 2.4 (0.2)	3.8 (0.3) 3.6 (0.2)
	Valo X (5 s, 2102 mW/cm^2^) (20 s, 1041 mW/cm^2^)	Class I Class V	2.2 (0.1) 1.1 (0.2)	2.5 (0.2) 2.1 (0.2)	Valo X (5 s, 2102 mW/cm^2^) (20 s, 1041 mW/cm^2^)	Class I Class V	2.2 (0.1) 1.7 (0.1)	2.6 (0.2) 2.2 (0.2)
		Class I Class V	4.1 (0.3) 2.4 (0.1)	3.9 (0.1) 2.9 (0.2)		Class I Class V	3.8 (0.4) 2.5 (0.3)	4.1 (0.1) 3.5 (0.1) 3.1 (0.1) 3.3 (0.1)
	SmartLite Pro (20 s, 1064 mW/cm^2^)	Class I Class V	2.9 (0.3) 2.4 (0.1)	2.9 (0.1) 3.1 (0.2)	SmartLite Pro (20 s, 1064 mW/cm^2^)	Class I Class V	2.7 (0.1) 2.3 (0.2)	
Miranda et al. (2024) [8, 17]	Polywave (3 s, 3200 mW/cm^2^)	TPFlow						
		9.16 (0.7)						
	Polywave (10 s, 1200 mW/cm^2^)	FBF						
		5.52 (1.3)						
Odum et al. (2023) [18]	Demi Ultra PLS (10 s, 1100 mw/cm^2^) (20 s, 1100 mw/cm^2^)	Resin						
		Tmax 10.4 (±0.1) Tmax 16.6 (±0.3)						
	VALO Grand (3 s, 3200 mW/cm^2^) (6 s, 3200 mW/cm^2^)	Tmax 10.4 (±0.1) Tmax 16.6 (±0.3)						
	Bluephase PowerCure (3 s, 3000 mW/cm^2^) (5 s, 2000 mW/cm^2^)	Tmax 12.0 (±0.1) Tmax 13.2 (±0.1)						
Thanoon et al. (2024) [19]	Monet Laser (1 s, 4800 mW/cm^2^) (3 s, 4800 mW/cm^2^)	Resin	TPFill			Resin	TEC	
		0 mm 1 mm 2 mm 3 mm 4 mm	1 s 7.1 (0.3) 16.7 (0.6) 16.6 (0.5) 14.8 (0.8) 12.7 (0.9) 3 s 11.4 (0.8) 20.6 (1.1) 20.8 (0.8) 19.9 (0.8) 18.4 (0.7)		Monet Laser (1 s, 4800 mW/cm^2^) (3 s, 4800 mW/cm^2^)	0 mm 1 mm 2 mm 3 mm 4 mm	1 s 5.1 (1.3) 11.2 (0.9) 9.5 (0.7) 7.5 (0.7) 5.9 (0.7) 3 s 11.5 (2.2) 18.5 (1.7) 16.4 (1.6) 13.5 (1.5) 11.3 (1.4)	
	PinkWave (3 s, 1700 mW/cm^2^) (20 s, 1600 mW/cm^2^)	0 mm 1 mm 2 mm 3 mm 4 mm	3 s 6.3 (0.8) 15.4 (0.7) 15.1 (0.9) 13.4 (0.6) 11.5 (0.4) 20 s 14.4 (1.2) 22.5 (0.5) 22.2 (0.6) 20.9 (0.5) 19.3 (0.3)		PinkWave (3 s, 1700 mW/cm^2^) (20 s, 1600 mW/cm^2^)	0 mm 1 mm 2 mm 3 mm 4 mm	3 s 7.4 (0.7) 12.4 (1.2) 11.6 (0.6) 10.3 (0.3) 8.8 (0.6) 20 s 15.1 (1.9) 21.3 (1.0) 21.6 (0.4) 20.4 (0.5) 18.8 (0.4)	
	Elipar S10 (5 s, 1600 mW/cm^2^) (20 s, 1080 mW/cm^2^)	0 mm 1 mm 2 mm 3 mm 4 mm	5 s 9.2 (1.7) 18.9 (1.2) 19.8 (1.0) 18.9 (0.8) 17.1 (0.7) 20 s 14.5 (1.8) 3.4 (2.2) 23.4 (1.7) 22.3 (1.9) 20.8 (2.0)		PinkWave (3 s, 1700 mW/cm^2^) (20 s, 1600 mW/cm^2^)	0 mm 1 mm 2 mm 3 mm 4 mm	5 s 9.2 (1.2) 17.8 (0.7) 16.7 (0.3) 14.2 (0.3) 11.9 (1.0) 20 s 15.2 (2.1) 22.9 (1.5) 21.1 (1.4) 19.6 (0.3) 16.8 (0.7)	

Abbreviations: AF, Admira Fusion X‐Tra; BBF, Beautifil Bulk Fill Flow; FBF, Filtek Bulk Fill Flowable; OBF, Filtek One Bulk Fill; PFill, Tetric PowerFill; PFlow, Tetric PowerFlow; VC, Viscalor; TEC, Tetric EvoCeram Bulk Fill.

### 3.4. Risk of Bias

Most included studies presented low methodological quality, with only one classified as having a moderate risk of bias. The remaining studies were rated as having a high risk of bias, with only one to three of the nine criteria met. Main concerns included a lack of tooth randomization, inadequate standardization of enamel/dentin surfaces, absence of single‐operator temperature analysis, no examiner blinding, and missing sample size calculation. Table 4 summarizes the risk of bias assessment.

**Table 4:** Risk of bias assessment.

**Study**	**Teeth randomization**	**Teeth free of caries**	**Standardization of enamel/dentine surface samples**	**Temperature analysis by a single operator**	**Blinding of the examiner**	**Sample size calculation**	**Complete outcome data**	**Risk of bias**
Wang et al. (2021) [14]	N	N	N	Y	Y	Y	Y	Medium
Yang et al. (2021) [15]	N	Y	N	N	N	N	Y	High
Maucoski et al. (2023) [16]	N	Y	N	N	N	N	Y	High
Miranda et al. (2024) [8, 17]	N	Y	N	Y	N	N	Y	High
Odum et al. (2023) [18]	N	N	N	N	N	N	N	High
Thanoon et al. (2024) [19]	N	N	N	N	N	Y	Y	High

### 3.5. Meta‐Analysis

Only three studies were considered suitable for inclusion in the meta‐analysis. The study by Odum et al. [18] was excluded because it did not distinguish between the control and intervention groups. The study by Thanoon et al. [19] was also excluded due to incompatibility in LCU power compared to other studies. Thus, the meta‐analysis included data from two studies evaluating the following comparisons: (i) light curing for 3 versus 10 s and (ii) light curing for 3 versus 20 s.

#### 3.5.1. Comparison: Light Curing of 3 and 10 s

**Figure 2:**
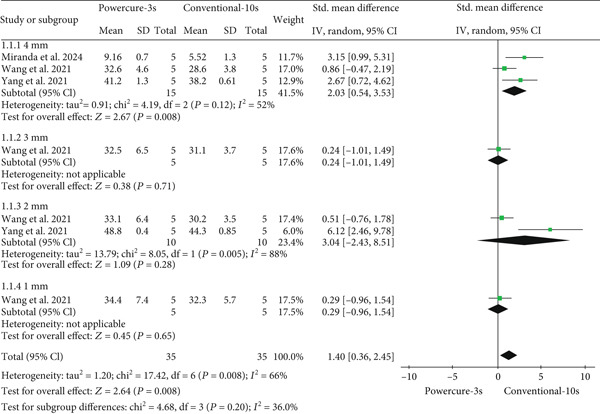
Forest plot presenting standardized mean differences and standard errors of temperature assessment between light curing of 3 and 10 s.

Figure 2 presents the forest plot comparing the temperature increase between the 3‐s (PowerCure system) and the conventional 10‐s light curing. The analysis showed a significant temperature increase with the 3‐s curing (*p* = 0.008), with moderate heterogeneity (*I* ^2^ = 66*%*). Subgroup analysis by increment thickness showed for 4 mm (*p* = 0.008; SMD = 2.03 [95% CI 0.54–3.53]), a significant difference favoring higher temperatures in 3‐s curing; for 3 mm (*p* = 0.71; SMD = 0.24 [95% CI −1.01 to 1.49]), no significant difference; and for 2 mm (*p* = 0.28; SMD = 3.04 [95% CI −2.43 to 8.51]) and for 1 mm (*p* = 0.65; SMD = 0.29 [95% CI −0.96 to 1.54]), both without significant differences. Overall, a greater temperature rise was observed with the 3‐s PowerCure protocol.

#### 3.5.2. Comparison: Light Curing of 3 and 20 s

**Figure 3:**
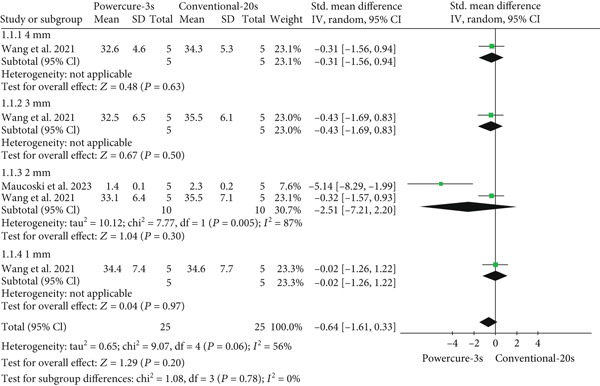
Forest plot presenting standardized mean differences and standard errors of temperature assessment between light curing of 3 and 20 s.

Figure 3 shows the forest plot comparing the temperature increase between 3‐ and 20‐s curing. No significant difference was observed (*p* = 0.20), with moderate heterogeneity (*I* ^2^ = 56*%*). Subgroup results by thickness were as follows: 4 mm (*p* = 0.63; SMD = −0.31; 95% CI [−1.56, 0.94]), 3 mm (*p* = 0.50; SMD = −0.43; 95% CI [−1.69, 0.83]), 2 mm (*p* = 0.30; SMD = −2.51; 95% CI [−7.21, 2.20]), and 1 mm (*p* = 0.97; SMD = −0.02; 95% CI [−1.26, 1.22]). These results indicate no statistically significant temperature difference across the thicknesses evaluated.

### 3.6. Quality of Evidence

Using the GRADE tool, the certainty of evidence for the 3 versus 10 s and 3 versus 20 s light‐curing comparisons was rated as very low. Downgrades were made for inconsistency (serious), due to high heterogeneity in study design, sample sizes, and temperature assessment methods, and indirectness (severe), since one study′s population did not provide sufficiently direct evidence. Detailed findings are shown in Table 5.

**Table 5:** Quality of the evidence—GRADE assessment.

**Certainty assessment—temperature rise**									
**Parameter**	**No. of studies**	**Study design**	**Risk of bias**	**Inconsistency**	**Indirectness**	**Imprecision**	**Other considerations**	**Important**	**Certainty**
3 and 10 s	3	Non RCT	Not serious^a^	Serious^b^	Serious^c^	Not serious^d^	None		⊕*ΟΟΟ* Very Low
3 and 20 s	2	Non RCT	Not serious^a^	Serious^b^	Serious^c^	Not serious^d^	None		⊕*ΟΟΟ* Very Low

^a^All included studies presented a moderate risk of bias.

^b^Presence of substantial heterogeneity.

^c^Indirectness is judged based on population, intervention, comparison, and outcome across studies, with the population probably not presenting sufficiently direct evidence.

^d^Evidence has not been rated down by any level.

## 4. Discussion

According to this systematic review, the null hypothesis—that high‐intensity light curing would not cause a temperature increase in bulk‐fill RBCs compared to standard protocols—must be rejected. Among the six included studies, four [14, 17–19] reported a significant rise in intrapulpal temperature associated with high‐intensity light exposure. These findings, derived from in vitro experiments, suggest that under specific clinical conditions, particularly in deep cavity restorations, high‐intensity curing protocols may generate heat levels sufficient to compromise pulpal health. However, it is important to stress that in vitro models lack biological thermoregulatory mechanisms, such as pulpal blood flow, and may therefore overestimate the actual clinical risk.

Bulk‐fill RBCs were developed to simplify clinical workflows by allowing the placement and curing of increments up to 4 mm thick in reduced time [9]. However, concerns persist regarding the potential thermal hazards of high‐intensity light curing, especially concerning pulpal safety [10]. Heat generated during polymerization originates from both the exothermic reaction of the resin composite and the energy output from the LCU [23], with irradiance, exposure time, and material thickness as key influencing factors [24, 25].

This review′s findings highlight that temperature elevation within the pulp chamber is proportional to the irradiance applied, even when using bulk‐fill RBCs engineered for rapid polymerization [14, 19]. Newer bulk‐fill RBCs incorporate reversible addition–fragmentation chain transfer (RAFT) mechanisms, which aim to mitigate polymerization stress and improve thermal behavior [26, 27]. However, evidence shows that temperature rises can still exceed critical thresholds under certain conditions.

An intrapulpal increase of more than 5.5°C may result in irreversible pulp damage [11]. Temperatures above 42.5°C are associated with thermal necrosis [28, 29]. While Maucoski et al. [16] reported that temperature elevations under high‐intensity curing remained below critical values, their findings were obtained under in vitro conditions lacking biological thermoregulatory mechanisms, such as pulpal blood flow [30]. Although some studies reported that intrapulpal temperature elevations under high‐intensity curing remained below critical thresholds, this safety margin is highly conditional. The actual risk is strongly influenced by clinical factors such as cavity depth, residual dentin thickness, and the positioning of the LCU. Emphasizing this context‐dependent nature is essential to avoid overgeneralization of laboratory findings to clinical practice.

Residual dentin thickness is a critical protective factor against thermal insult. In vitro studies indicate that a dentin layer of at least 1 mm effectively insulates the pulp [15–17]. However, Miranda et al. [17] showed that a 0.5‐mm dentin thickness resulted in significantly greater temperature rises under high‐intensity curing, increasing the risk of pulpal injury in clinical cases involving deep cavities, thin dentin walls, or compromised pulpal health. This aligns with in vivo findings by Runnacles et al. [31], who demonstrated a positive correlation between higher radiant emittance and increased intrapulpal temperature.

Although the exact threshold for thermal injury to the pulp remains debated, there is consensus that intrapulpal temperature rises must be minimized [32]. Despite its vascularization, the pulp remains vulnerable to excessive heat, which can lead to hyperalgesia, spontaneous pain, or even pulpitis [30, 33]. Despite advances in resin formulation, in vitro studies still demonstrate significant heat generation under high‐irradiance curing, particularly when dentin thickness is limited [14, 17, 18, 34]. Photoinitiators such as AFCT help regulate exothermic heat during polymerization [35]. When combined with RAFT technology [26, 27], they improve conversion degree and reduce shrinkage [26, 33].

Although many reported temperatures remained below clinical thresholds [16], these in vitro results lack the complexity of real tissue environments, notably the absence of heat dissipation through pulpal blood flow [30]. The heightened temperatures recorded with reduced dentin thickness [15, 17] reinforce the clinical importance of maintaining adequate residual dentin during restorative procedures.

Several strategies have been proposed to mitigate thermal risk: (1) Air cooling has shown significant potential in reducing intrapulpal temperature, particularly in deep cavities or with thin dentin [36, 37]; (2) intermittent or pulse curing significantly lowers temperature rise while maintaining adequate polymerization [38]; (3) modifying exposure time and accounting for RBC shade can also control heat generation, especially in deep restorations [39]; and (4) using RBCs specifically designed for high‐intensity curing with optimized photoinitiators (e.g., RAFT and AFCT) is essential to prevent incomplete polymerization or excessive thermal output [26, 35, 40].

Correct positioning of the LCU tip is essential to control heat generation and ensure effective polymerization. AlShaafi [41] highlighted that both improper angulation and excessive distance of the LCU can cause uneven light distribution, incomplete curing, higher residual monomer release, and greater thermal effects. Similarly, Duratbegović et al. [42] demonstrated that variations in the distance between the LCU tip and the composite surface significantly affect curing efficiency and intrapulpal temperature. Taken together, these findings emphasize that the LCU tip should be positioned as close as possible and strictly perpendicular to the restoration surface, avoiding oblique angulation, to optimize polymerization and minimize intrapulpal heating.

The clinical relevance of this systematic review lies in emphasizing the need for individualized light‐curing strategies based on clinical conditions, residual dentin thickness, and RBC properties. While rapid, high‐intensity curing protocols can enhance workflow efficiency, they should be applied cautiously to avoid compromising pulp health.

This review has several limitations. Considerable heterogeneity was observed across the included studies in terms of sample type, measurement methods, and light‐curing protocols, which reduced the certainty of evidence. Moreover, all studies were conducted in vitro. Despite their value, laboratory models lack the biological complexity of the oral environment, particularly thermoregulatory mechanisms like pulpal perfusion. Variability in experimental designs, baseline temperatures, and assessment methods also contributed to the heterogeneity observed. To enhance evidence quality and clinical applicability, future research should address these points: (1) Develop standardized in vitro models simulating pulpal perfusion; (2) conduct well‐designed clinical trials focusing on high‐intensity curing in deep cavities with minimal residual dentin; (3) investigate long‐term biological outcomes, including pulp vitality and inflammatory markers; (4) explore synergistic cooling strategies, such as continuous airflow during curing; (5) evaluate different LCU designs, spectral outputs, and resin composite polymerization kinetics; and (6) analyze the interaction between RBC viscosity, filler content, and thermal profiles.

The findings of this systematic review indicate that high‐intensity light curing can significantly increase intrapulpal temperature compared with standard protocols, particularly in deep cavities with thin residual dentin. Although bulk‐fill RBCs and newer photoinitiators (RAFT and AFCT) improve polymerization efficiency and reduce stress, thermal rises may still approach or exceed critical thresholds under certain conditions. Residual dentin thickness remains the most important protective factor, with ≥ 1 mm providing effective insulation. Conversely, thicknesses ≤ 0.5 mm markedly increase the risk of pulpal injury. Adjunctive strategies such as air cooling, intermittent or pulse curing, and correct LCU positioning can mitigate thermal effects without compromising polymerization.

While most in vitro studies reported temperature elevations below irreversible thresholds, these results must be interpreted cautiously since laboratory conditions do not replicate biological thermoregulation. The clinical translation of these findings, therefore, requires well‐designed trials and standardized models incorporating pulpal perfusion. In conclusion, high‐intensity curing offers valuable efficiency gains but should be applied selectively and with protective measures to safeguard pulpal health. An individualized, evidence‐based approach remains essential for safe clinical use.

## 5. Conclusion

High‐intensity light curing results in an increased temperature in bulk‐fill RBCs, according to in vitro studies. Although the overall certainty of the evidence is very low, the technique can be considered safe when an adequate thickness of residual dentin is present and when the LCU tip is positioned directly over the restoration surface. However, in deep cavities with thin residual dentin thickness, high‐intensity curing should be avoided due to the higher risk of intrapulpal temperature rise. Clinicians should consider adjunctive measures to mitigate potential thermal damage and avoid postoperative sensitivity.

## Ethics Statement

As this was a systematic review, neither ethical approval nor consent to participate was needed.

## Disclosure

All authors have read and agreed to the published version of the manuscript.

## Conflicts of Interest

The authors declare no conflicts of interest.

## Author Contributions

S.B.M., M.R.S., and G.L.d.M. contributed to the study conception and design, and S.B.M. and L.A.S.F. contributed to the material preparation and data collection. M.J.G.S.B. and V.M.d.S.R. performed the statistical analysis. A.K.M.d.A., M.A.J.R.M., and R.B.E.L. critically revised the manuscript and supervised the review process. The first draft of the manuscript was written by S.B.M., M.R.S., and G.L.d.M.

## Funding

No funding was received for this manuscript.

## Data Availability

The datasets used and/or analyzed during the current study are available from the corresponding author.
